# The health benefits of alkaline water: is it a fact or marketing myth?

**DOI:** 10.3389/fmed.2026.1849818

**Published:** 2026-06-26

**Authors:** Hassan Alebrahim, Adnan Alsaei, Peter Sefen, Mohammed Hasan, Abdulmuhsen Ramadhan, G. Roshan Deen

**Affiliations:** Materials for Medicine Research Group, School of Medicine, Royal College of Surgeons in Ireland (RCSI), Medical University of Bahrain, Busaiteen, Bahrain

**Keywords:** alkaline water, bone health, diabetes, gastro intestinal, health benefits, myth or fact

## Abstract

**Background:**

Alkaline water has gained widespread popularity and is frequently marketed for benefits related to hydration, acid–base balance, metabolic health, and immune function. These claims are increasingly encountered in primary care, yet their clinical validity remains uncertain.

**Objective:**

To critically evaluate current evidence on alkaline water, focusing on its physiological effects, clinical relevance, and safety in routine patient care.

**Methods:**

A narrative review of the literature was conducted, including human clinical studies, experimental interventions, and animal models. Outcomes examined included gastrointestinal health, hydration status, bone metabolism, metabolic parameters, oxidative stress, and immune function.

**Results:**

Evidence suggests that alkaline water provides limited, context-specific benefits. The most consistent findings relate to gastrointestinal effects, particularly pepsin inactivation and potential improvement in reflux-related symptoms. Modest effects on hydration markers, urinary pH, and selected indicators of bone resorption have been reported, especially with bicarbonate-rich formulations. In contrast, evidence supporting metabolic, antioxidant, and immunological benefits is inconsistent and largely derived from small or preclinical studies. Claims of systemic alkalinization, enhanced immunity, anti-aging, and disease prevention are not supported by robust clinical evidence.

**Clinical implications:**

Alkaline water should not be routinely recommended in primary care. Clinicians should emphasize evidence-based interventions and exercise caution in vulnerable populations, including individuals with chronic kidney disease, those on acid-suppressive therapy, pregnant women, and children.

**Conclusion:**

Alkaline water has limited clinical utility. Its effects are modest and likely driven more by mineral composition than pH alone. Further well-designed, long-term studies are required before definitive recommendations can be made.

## 1 Introduction

Over the last decade, alkaline water has evolved from a niche wellness product into a widely recognized global health trend, promoted extensively for its purported ability to enhance a range of physiological functions. Characterized by an elevated pH, typically above 8.0, and modified through mineral enrichment or electrolysis, alkaline water is claimed to improve hydration, optimize metabolic processes, neutralize systemic acidity, support bone mineralization, and provide antioxidant effects. These claims have gained considerable traction through intensive marketing campaigns and social media dissemination, despite the limited availability of strong scientific evidence. The rapid growth of the alkaline water market reflects a broader modern interest in lifestyle-based approaches to health optimization and emphasizes the need to critically evaluate such claims through an evidence-based framework.

From a physiological perspective, acid base homeostasis is tightly regulated by renal, pulmonary, and buffering systems that maintain arterial pH within the narrow range of 7.35–7.45. Even minor deviations from this range can affect enzymatic activity, cellular metabolism, and oxygen transport. Supporters of alkaline water argue that modern diets rich in processed carbohydrates, animal proteins, and acidic beverages impose a chronic acid load that challenges these regulatory mechanisms. They suggest that alkaline water consumption may reduce dietary acidity and help restore internal pH balance. However, this proposition remains controversial. Current evidence indicates that in healthy individuals, acid base equilibrium is effectively preserved except in pathological conditions such as renal insufficiency or diabetic ketoacidosis (1).

Despite ongoing scientific skepticism, numerous experimental and clinical studies have explored whether alkaline water may exert measurable health effects. For example, Koufman and Johnston (2) showed that water with a pH of 8.8 irreversibly denatures pepsin, suggesting possible therapeutic value in laryngopharyngeal reflux. Similarly, Meunier et al. (3) reported that consumption of high-calcium mineral water lowered biochemical indices of bone remodeling in postmenopausal women with low calcium intake, indicating potential effects of mineral-water composition on skeletal metabolism. Other studies and reviews have examined its effects on hydration status, blood viscosity, antioxidant capacity, inflammatory markers, and metabolic parameters in both athletic and clinical populations. However, the results have remained inconsistent and are often limited by small sample sizes, heterogeneous methodologies, and short intervention periods (4–6).

Given the widespread promotion of alkaline water, it is essential to distinguish scientifically supported findings from marketing exaggeration. While limited evidence suggests modest benefits in specific settings, especially in reflux management and minor improvements in hydration, claims regarding systemic alkalinization, immune enhancement, cancer prevention, or anti-aging effects remain unsupported. In addition, concerns about long term safety, particularly in relation to high pH or electrolyzed water, require further investigation. Chronic consumption may alter gastrointestinal acidity, nutrient bioavailability, and renal function, highlighting the need for more research to establish appropriate safety guidance.

In light of these considerations, the present review aims to critically evaluate the scientific validity of the claimed health benefits of alkaline water particularly in gastrointestinal health and to examine the physiological and clinical risks associated with its prolonged use. By synthesizing current peer reviewed literature, this review seeks to determine whether alkaline water represents a verifiably effective health intervention or whether its popularity primarily reflects persuasive marketing, anecdotal experience, and the appeal of simple solutions to complex biological processes.

## 2 Understanding alkaline water

### 2.1 Definition and properties (pH, mineral content, production methods)

Alkaline water is defined as drinking water with a pH greater than 7, typically ranging from 8 to 10, in contrast to neutral water with a pH of 7 or mildly acidic tap water. Its elevated pH may occur naturally through chemical interactions between groundwater and carbonate rich rocks, resulting in dissolution of bicarbonate together with calcium, magnesium, and potassium ions. It may also be produced artificially through ionization or electrolysis devices that separate water into alkaline and acidic fractions, or through mineral addition systems that enhance buffering capacity (7, 8). Bicarbonate rich mineral waters generally exhibit greater alkalinity than low mineral waters and may influence urinary pH and hydration markers without altering tightly regulated blood pH (4, 9).

Artificially produced alkaline water, often marketed as alkaline ionized water (AIW) or electrolyzed reduced water (ERW), frequently has a negative oxidation reduction potential (ORP), which has been promoted as an indicator of antioxidant potential (10). In athletes, the consumption of mineral rich high pH water has been associated with modest increases in urinary pH, suggesting transient renal buffering rather than sustained systemic alkalinization (11). Meunier et al. (3) demonstrated that high-calcium mineral water lowered biochemical indices of bone remodeling in postmenopausal women with low calcium intake, suggesting that mineral composition, rather than pH alone, may be important in shaping skeletal effects. Nevertheless, blood pH homeostasis in healthy individuals prevents any clinically meaningful change in blood pH following alkaline water consumption, maintaining the normal physiological range of approximately 7.35–7.45 (12).

Alkaline water may also occur naturally through geological processes, particularly when water passes through mineral rich rocks such as limestone or volcanic formations, leading to increased bicarbonate and alkaline mineral content. These interactions influence the water’s pH and contribute to its distinct chemical profile and potential health related properties. As shown in Table 1, several natural water sources across different geographical regions display varying levels of alkalinity and distinct mineral characteristics.

**Table 1:** Natural alkaline water (pH 8–10) across different geographical regions.

Country	Name of water	pH	Special characteristics	References
Saudi Arabia	Zamzam water	8.0	Naturally alkaline; mineral-rich; reported Ca and K content; trace Li also noted	(13)
Portugal	Monchique	9.4	Still natural mineral water; Na–HCO₃ type	(14)
Japan	Hita Tenryosui water	8.3	Natural reduced water; slightly alkaline; active hydrogen discussed in the paper	(15)
China	Changbaishan Fall water	8.3	Natural waterfall water from a volcanic area near Mt. Baekdu/Changbai	(16)

### 2.2 Differences between alkaline water, regular water, and other specialty waters

All potable water shares the same basic molecular structure, H₂O. However, waters differ considerably in chemical composition, buffering capacity, and physiological effects. Regular municipal or bottled drinking water generally falls within a near neutral pH range of 6.5 to 8.5, contains low total dissolved solids below 500 mg/L, and primarily functions to maintain hydration and thermoregulation (17, 18). Alkaline water, by contrast, typically has a pH between 8 and 10, a negative oxidation reduction potential, and a higher concentration of buffering minerals such as bicarbonate, calcium, magnesium, and potassium (19, 20). These constituents may neutralize acid loads *in vitro* and can transiently increase urinary pH *in vivo* without changing systemic blood pH (20). In one trial, consumption of a high mineral alkaline water after exhaustive exercise reduced post exercise blood viscosity and improved fluid retention compared with neutral water, suggesting small hydration related benefits under metabolic stress (21). However, randomized studies consistently conclude that these effects are modest and context dependent, emphasizing that alkalinity alone is not the sole determinant of improved hydration or acid base regulation (22).

The beverage market now includes a broad range of specialty waters such as natural mineral, artesian, ionized, hydrogen enriched, and oxygenated waters, each differing in mineral profile and production method. Natural mineral waters are obtained directly from underground sources and are characterized by a relatively stable ionic composition, often rich in bicarbonate and calcium. Some small clinical studies have linked these waters to improved bone metabolism and reduced renal acid excretion (20). Artificially ionized or electrolyzed waters are produced through electrolysis, which raises pH and lowers oxidation reduction potential, often using domestic auto ionizer machines. These machines operate by passing an electric current through platinum coated titanium electrodes, causing water molecules to dissociate. Alkaline forming cations migrate toward the cathode to form alkaline reduced water, while acid forming anions accumulate at the anode.

**Figure 1:**
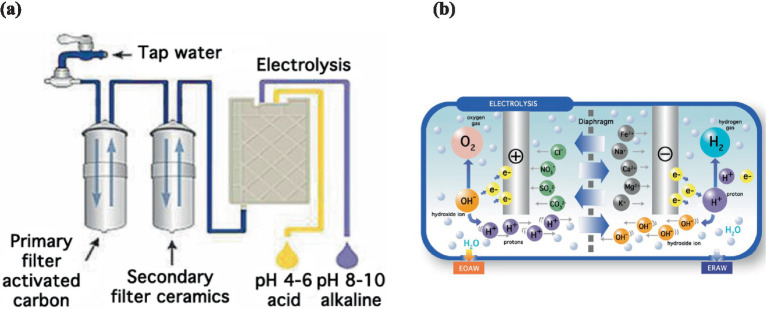
Electrolysis of water to generate alkaline water. **(a)** General overview of a commercial electrolyzed water generator. **(b)** General overview of a commercial electrolyzed water generator. Adapted from Henry and Chambron (23), “Physico-Chemical, Biological and Therapeutic Characteristics of Electrolyzed Reduced Alkaline Water (ERAW),” published in Water by MDPI, under the terms of the Creative Commons Attribution 3.0 License (CC BY 3.0).

Water ionizers are connected to the mains supply, where water is first filtered through activated carbon to reduce chloride levels and protect the electrolytic cell. The filtered water must contain a mineral content of at least 50 mg·L^−1^ to undergo electrolysis within a chamber containing platinum coated titanium electrodes separated by a semipermeable diaphragm, as shown in Figure 1a.

At the anode, acidic oxidized water is produced with a pH between 4 and 6 and a redox potential reaching up to +900 mV. As mineral ions such as bicarbonate and chloride accumulate, protons and oxygen are released according to the following reaction:


$$\mathrm{2H}₂O(\ell )\to O₂(g)+{\mathrm{4H}}^{+}(\mathrm{aq})+4\;e^{-}.$$

Conversely, alkaline reduced water is generated at the cathode, reaching a pH between 8 and 10 and a redox potential as low as −600 mV. Mineral cations such as sodium, potassium, calcium, and magnesium accumulate there while hydroxyl ions and hydrogen are produced, as seen in Figure 1b (23):


$$2\;H₂O(\ell )+2\;e^{-}⇄H₂(g)+2\;{\mathrm{OH}}^{-}(\mathrm{aq}).$$

This electrolysis process may also generate molecular hydrogen, although findings regarding antioxidant benefits remain inconsistent (24). Hydrogen infused waters, for example, may display temporary free radical scavenging activity but can lose effectiveness within hours of bottling (25). In addition, comparative analyses of commercial bottled waters have identified variability between labeled and measured mineral content or pH, highlighting the need for improved quality control and accurate product labeling (22, 26). Pitale et al. compared the pH of different brands of alkaline water and concluded that five out of six alkaline water brands exhibited mean pH levels lower than that indicated on their commercial labels (25).

Overall, alkaline water and other specialty waters differ from regular water mainly in mineral content and commercial claims, yet the clinical significance of these differences remains uncertain. The body’s acid base homeostasis continues to be regulated primarily by renal and respiratory mechanisms rather than by the pH of ingested water (12).

### 2.3 Digestion of alkaline water

Alkaline water is not “digested” like food because it contains no nutrients that need enzymatic breakdown. After swallowing, it passes through the esophagus into the stomach, where it mixes with gastric acid. Its higher pH may temporarily raise the stomach pH, but the stomach usually restores its acidic environment by secreting more acid. In people with reflux, alkaline water may have a specific digestive benefit because pH 8.8 alkaline water was shown *in vitro* to inactivate pepsin, an enzyme that can irritate the esophagus and throat during reflux, and it also showed acid-buffering capacity. However, this does not mean alkaline water changes the body’s overall pH or replaces treatment for reflux disease (2).

A study suggested that most interactions between alkaline water and the digestive tract lies not in the alkalinity or pH of water ingested, but the presence of Hydrogen molecules, where neutral and non-polar molecular Hydrogen can diffuse into plasma membranes, mitochondria, and nuclei at a high rate (27). Due to this high diffusivity and membrane permeability, hydrogen molecules can diffuse through the course of the gastrointestinal mucosa into local tissues and then into the local circulation, which can lead to downstream modulation of gene expression, signal transduction, and production of antioxidant enzymes.

However, recent evidence warns that a negative oxidation–reduction potential (ORP) is not a reliable proxy for dissolved molecular hydrogen (H2), as ORP is heavily influenced by pH and other physicochemical conditions. Consequently, studies or commercial claims relying solely on pH or ORP without directly measuring H2 concentration must be interpreted critically. Additionally, this study highlights the widespread, inaccurate use of ORP meters as “H2 meters” as they measure water alkalinity and redox potential rather than actual hydrogen content, which falsely overestimates H2 levels in high-pH commercial water. This distinction is critical because H2 is a light, highly diffusive gas that easily escapes into the atmosphere during the manufacturing and bottling of alkaline water. As a result, commercial hydrogen claims are often exaggerated, and the actual H2 content in bottled water may fall short of the therapeutic concentrations used in successful clinical trials, ultimately misleading consumers. This makes it difficult to conclude from clinical trials that measured positive outcomes can be applied to all commercial products (27).

### 2.4 Production methods, quality control, and regulatory perspectives

The commercial growth of alkaline water has been driven by both natural mineral sources and technological developments that allow large scale artificial production. Naturally alkaline waters originate in carbonate and silicate bearing aquifers, where prolonged water rock interactions enrich groundwater with bicarbonate and alkaline earth metals such as calcium, magnesium, and potassium (23). These natural sources are often protected under environmental regulations to preserve mineral stability and prevent contamination. In contrast, artificially produced alkaline waters, including electrolyzed reduced water (ERW) and alkaline ionized water (AIW), are generated through electrolysis or ion exchange processes that separate tap water into alkaline and acidic streams (24). Electrolysis modifies both pH and oxidation reduction potential, producing waters that may reach a pH of 10 and display negative ORP values. However, the final output depends strongly on source water composition, electrode material, voltage, and duration of exposure, which can create substantial variation in quality and reproducibility between brands (25, 26).

Some manufacturers also use post treatment mineralization, such as adding calcium carbonate or magnesium oxide, to stabilize pH or improve taste. However, this may also increase total dissolved solids beyond recommended thresholds (26). Laboratory analyses suggest that although electrolysis-based waters may transiently increase urinary pH, their mineral concentrations often remain below physiologically meaningful levels, implying that many commercial alkaline products may rely more on marketing appeal than on substantial compositional differences (25, 28). Excessive alkalinity with pH above 11 or contamination during production may cause gastrointestinal irritation, while low quality electrodes in home ionizers may release nickel or platinum nanoparticles, raising possible toxicity concerns. Regulatory agencies such as the World Health Organization (WHO), the U. S. Environmental Protection Agency (EPA), and the European Food Safety Authority (EFSA) therefore require regular monitoring of mineral concentrations, microbial purity, and container safety (29). In the European Union, naturally alkaline mineral waters may be classified as therapeutic if they contain more than 600 mg/L bicarbonate or more than 1,500 mg/L total dissolved solids, whereas artificially ionized waters are categorized as processed beverages without medicinal recognition (26).

From an environmental and sustainability perspective, the alkaline water industry has also been criticized for extensive plastic bottle use, energy intensive electrolysis, and the carbon footprint associated with global distribution. These concerns have encouraged pilot initiatives that promote onsite ionization systems and recyclable aluminum packaging to reduce waste. Taken together, these observations show that although advances in electrochemical water treatment and mineral enhancement have broadened consumer options, strict quality control, accurate labeling, and sustainable production practices remain essential to ensure that the claimed benefits of alkaline water are scientifically credible, safe, and environmentally responsible (30).

## 3 Digestive health and acid reflux

**Figure 2:**
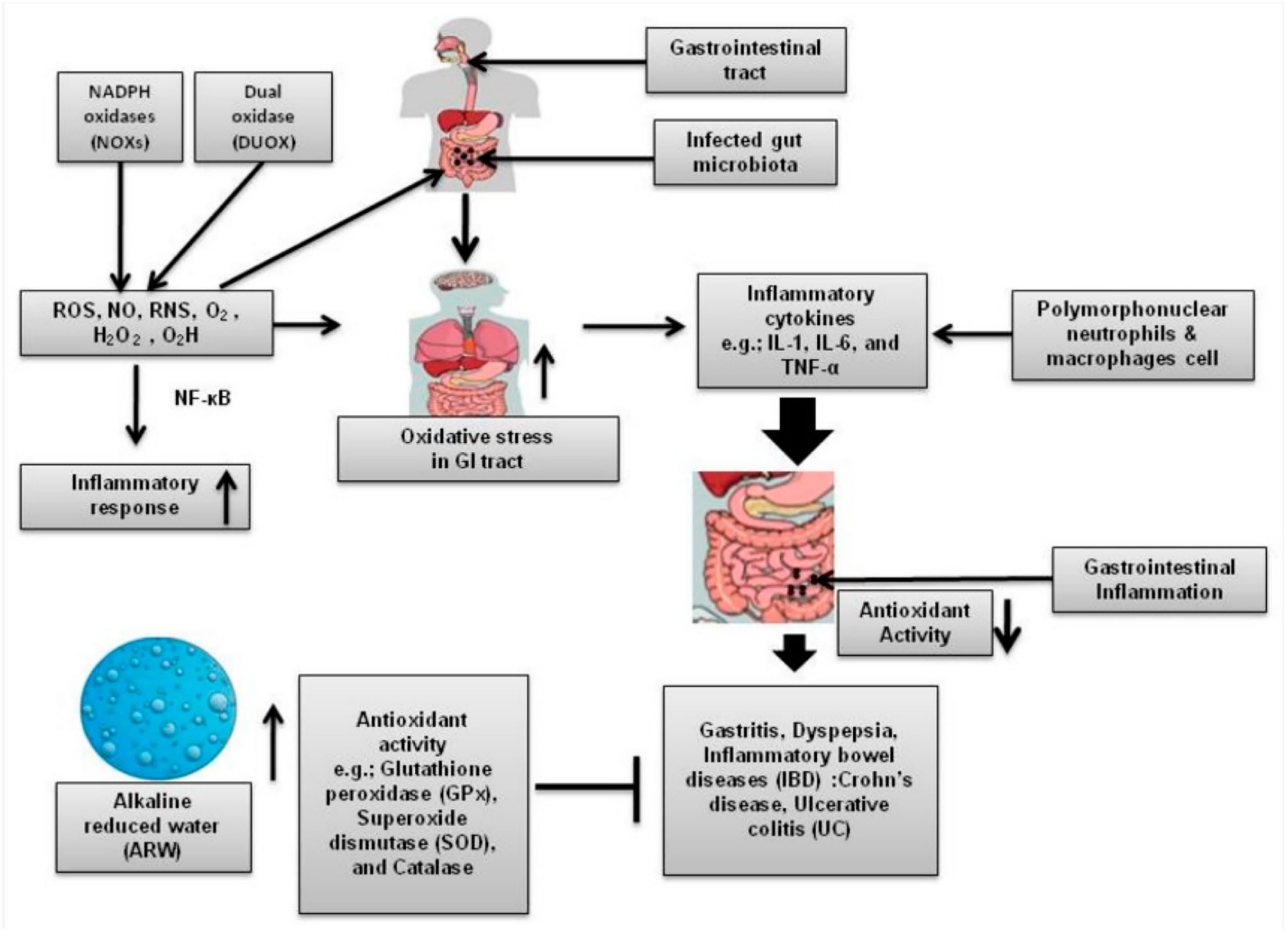
Role of oxidative stress and inflammation in GI diseases and effect of ARW as a potential candidate in GI diseases due to its antioxidative and anti-inflammatory actions. ARW, Alkaline reduced water; ROS, Reactive oxygen species; NO, Nitric oxide; RNS, Reactive nitrogen species; O₂H, Hydroperoxyl radical; O₂, Superoxide; H₂O₂, Hydrogen peroxide; NF-κB, Nuclear factor-kappa B; TNF-*α*, Tumor necrosis factor alpha; IL, Interleukin; GPx, Glutathione peroxidase; SOD, Superoxide dismutase; IBD, Inflammatory bowel diseases; UC, Ulcerative colitis; NOXs, NADPH oxidases; DUOX, Dual oxidase. Reprinted from Bajgai et al. (31), “Effects of Alkaline-Reduced Water on Gastrointestinal Diseases,” published in Processes by MDPI, under the terms of the Creative Commons Attribution 4.0 International License (CC BY 4.0).

Some experimental studies suggest that alkaline reduced water or hydrogen-rich water may have antioxidant and anti-inflammatory effects in gastrointestinal disease models. However, these effects should not be attributed to alkalinity alone, as dissolved molecular hydrogen and mineral composition may be important contributors. These proposed mechanisms and pathways are illustrated in Figure 2.

### 3.1 Gut microbiota and oxidative environment

The gastrointestinal tract contains a major portion of the body’s commensal microbiota, which plays an essential role in regulating enzymatic activity, oxidation, and immune homeostasis (32). The fermentation of fiber and resistant starch is carried out by beneficial bacteria such as *Bifidobacteria*, *Prevotella*, and lactobacilli, all of which are strict anaerobes. This process produces short chain fatty acid metabolites such as butyrate, propionate, and acetate, which support intestinal barrier integrity, mucous secretion, immune regulation, and overall gut homeostasis (33, 34).

This contributes to a physiologically low oxidation state in the gut, often described as an environment with low oxidation reduction potential, which supports the growth of strict anaerobes (31). Such a low oxidation environment is important because it prevents the rise in oxidative stress that commonly accompanies inflammation. However, in conditions of high oxidation reduction potential, the presence of oxygen in the form of reactive oxygen species increases. Excessive reactive oxygen species can damage proteins, nucleic acids, and lipids, while also promoting apoptosis and necrosis (35). They are also harmful to strict anaerobes, as these bacteria are adapted to oxygen deprived environments and may sustain DNA damage when exposed to oxidative stress (36).

In contrast, oxidative environments favor the growth of pathogenic bacteria such as *Salmonella* and *E. coli*, which are opportunistic facultative anaerobes capable of utilizing reactive oxygen species and thriving under oxidative conditions. In the presence of reactive oxygen species, these organisms shift to efficient aerobic respiration, giving them a competitive advantage over beneficial obligate anaerobes and allowing them to replace them. As a result, microbial dysbiosis and increased oxidation develop, both of which are predictors of gut injury, inflammation, and chronic gastrointestinal disease (37–39).

### 3.2 Enzymatic denaturation and buffering effects of alkaline water

Alkaline reduced water with a pH of 8 or above has been reported in several studies to improve the symptoms and progression of gastrointestinal disorders such as gastroesophageal reflux disease, peptic ulcer disease, and dyspepsia. One study demonstrated that alkaline reduced water with a pH of 8.8 irreversibly denatures pepsin 3b at a concentration of 1.5 mg/mL, similar to levels found in the stomach, and reduces the rate of hydrolysis of a synthetic peptide substrate by approximately fivefold compared with tap water. This indicates a marked reduction in pepsin activity in alkaline reduced water samples (2, 40).

Koufman and colleagues also showed that the high pH of alkaline reduced water provides buffering capacity, allowing it to resist small reductions in pH caused by hydrochloric acid. Hydrogen carbonate minerals are thought to bind directly to newly generated hydrogen ions, thereby limiting hydrochloric acid formation and reducing gastric acidity (41). Another review supported the use of alkaline reduced water as a treatment option for acid reflux because of its capacity to inactivate pepsin and reduce pepsin induced cellular injury within the gastrointestinal tract (42–44). Similarly, a systematic review that examined alternatives to antacid medications concluded that alkaline reduced water may be effective when combined with a low acid diet for the management of laryngopharyngeal reflux (45–49). Because of its high pH, negative oxidation reduction potential, and most importantly dissolved hydrogen content, one review also noted that the Japanese government recognized the health benefits of alkaline water in improving hyperacidity, indigestion, and chronic diarrhea, leading to the approval of household devices that generate alkaline reduced water as early as 1966. The same review further suggested potential benefits in suppressing extraintestinal oxidation related diseases such as diabetes and cancer (50).

### 3.3 Symptomatic and histological improvement

Further support for these claims comes from a randomized double-blind placebo-controlled pilot study showing that alkaline reduced water improved symptoms and quality of life scores in patients with diarrhea predominant irritable bowel syndrome after 8 weeks of intake (51). In addition, a systematic review of clinical trials reported strong evidence for symptom improvement in dyspepsia and gastroesophageal reflux disease. Two intervention studies from Germany compared heartburn symptoms before and after daily intake of 1.5 L of hydrogen carbonate rich mineral water for 6 weeks. One study demonstrated a reduction in the mean frequency of weekly heartburn episodes by 5.1 and a decrease in mean episode duration by 19 min, while the other showed a mean reduction of 4.8 episodes per week and a decrease in duration of 25.8 min. Trials from Italy and Russia included in the same review also found significant improvement in epigastric pyrosis and dyspeptic symptoms in patients with functional dyspepsia treated with alkaline reduced water (51). ARW also significantly prevented increases in epithelial cell apoptosis and DNA fragmentation, suggesting protective effects against acute gastric mucosal injury in rats (52).

**Figure 3:**
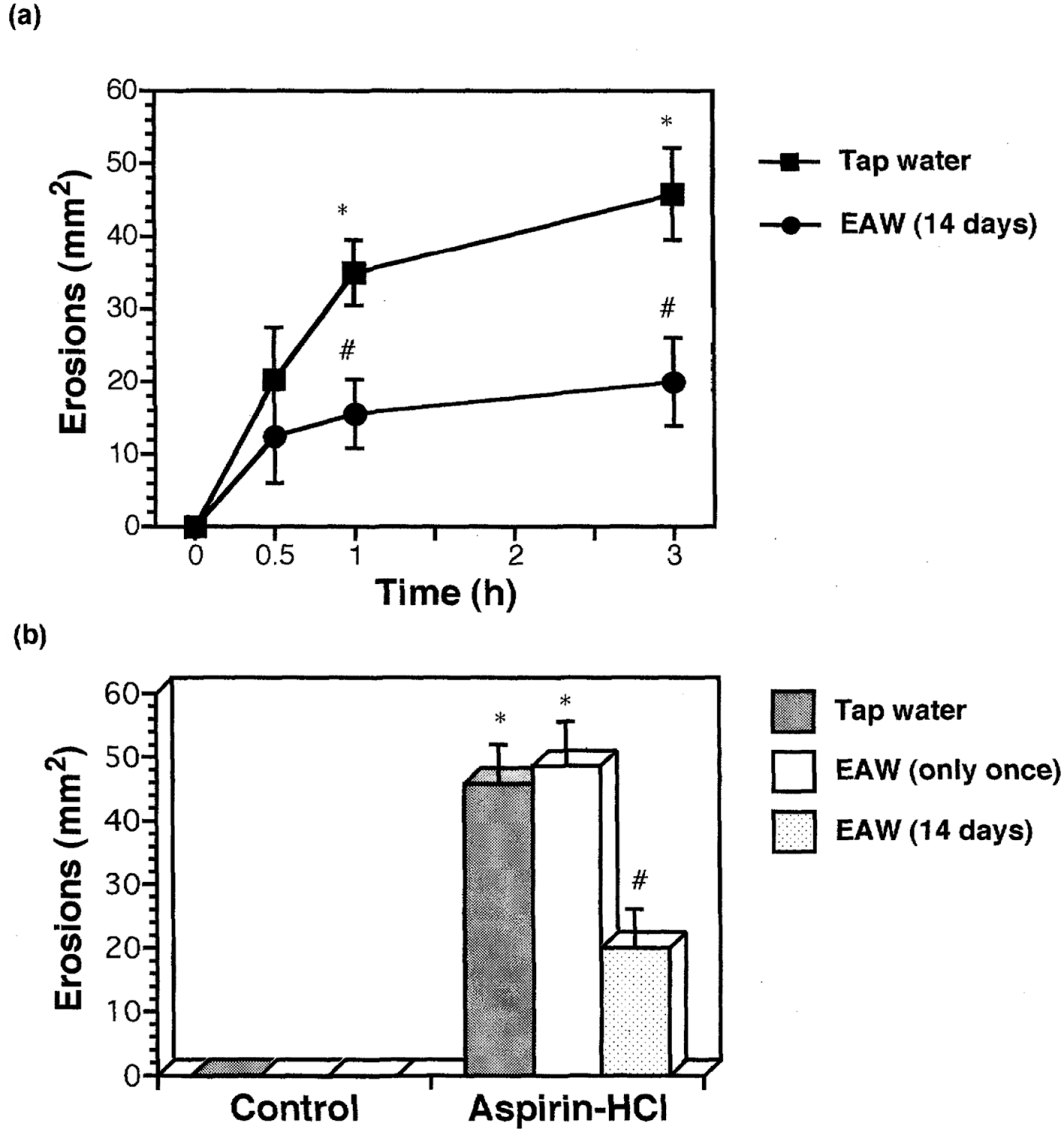
Time-course changes in the area of gastric erosions after the administration of aspirin-HCl **(a)** and the effects of chronic or acute administration of electrolyzed alkaline water (EAW) **(b)**. Treatment with EAW was performed by gastric intubation 1 h before aspirin administration or by providing a supply for free drinking for 14 days. Values are the mean ± SE for 6–8 rats. ^*^p < 0.01 when compared with control rats receiving tap water alone, and ^#^*p* < 0.01 when compared with rats receiving tap water plus aspirin-HCl. Reprinted from Naito et al. (52), “Chronic Administration with Electrolyzed Alkaline Water Inhibits Aspirin-Induced Gastric Mucosal Injury in Rats through the Inhibition of Tumor Necrosis Factor-α Expression”, © The Editorial Secretariat of JCBN, under the Creative Commons Attribution-NonCommercial-NoDerivatives License CC BY-NC-ND 4.0.

There is further evidence suggesting histological improvement in mucosal injury such as ulcers and erosions. One study found that treatment with alkaline reduced water for 2 weeks reduced the total area of aspirin-hydrochloric acid induced gastric lesions in rats by 57 percent, accompanied by reduced mucosal and submucosal neutrophilic infiltration, mucosal bleeding, and erosion, in comparison with tap water (52). These effects are demonstrated in Figures 3a,b.

These findings emphasize the broader therapeutic potential of alkaline reduced water in chronic gastritis and peptic ulcer disease, both of which are strongly linked to *Helicobacter pylori* infection. Research has also suggested possible anti-inflammatory and antioxidant effects in inflammatory bowel disease, including Crohn’s disease and ulcerative colitis. To understand these possible benefits, it is important to first examine the underlying pathology of these conditions (53–55).

### 3.4 Protective role of microbial metabolites

Under normal conditions, natural anti-inflammatory mechanisms protect the gut from injury. Short chain fatty acids (SCFAs) such as butyrate are bacterial metabolites used by colonocytes and have been shown to suppress pro inflammatory pathways by inhibiting nuclear factor kappa B signaling and histone deacetylase activity (56). Studies have also demonstrated a significant reduction in fecal SCFAs, particularly butyrate, in patients with inflammatory bowel disease due to severe dysbiosis, thereby contributing to ongoing inflammation and oxidative stress (57).

Consistent with this, a clinical trial investigating oral butyrate supplementation in rat models of colitis demonstrated a significant reduction in messenger RNA expression of pro inflammatory cytokines such as interleukin 6, tumor necrosis factor alpha, and interleukin 1 beta, together with inhibition of mitochondrial reactive oxygen species production (58). These findings underline the importance of a balanced gut microbiota and adequate SCFA production in limiting inflammation and oxidative stress. This helps identifying important therapeutic targets and supports the hypothesis that alkaline reduced water may provide restorative effects in inflammatory bowel disease, chronic gastritis, and peptic ulcer disease (59).

### 3.5 Anti-inflammatory, immunomodulatory, and antioxidative effects of alkaline water

**Figure 4:**
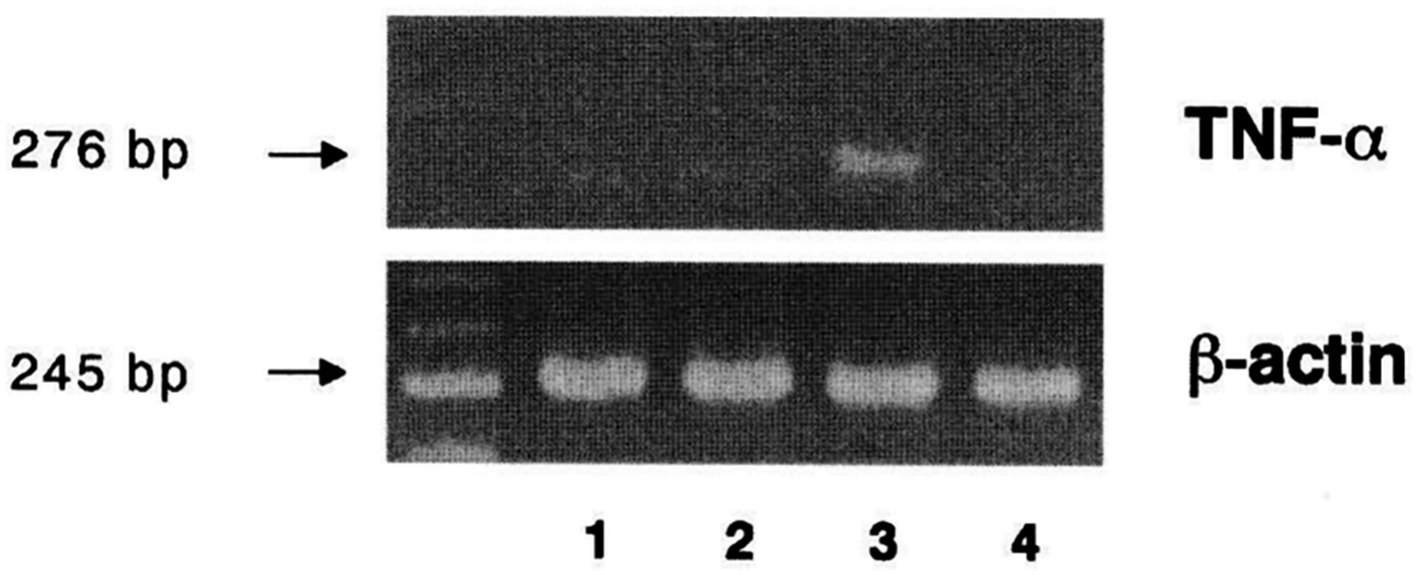
The effect of electrolyzed alkaline water (EAW) on the expression of tumor necrosis factor-a (TNF-a) mRNA after aspirin-HCl administration. A representative 2% agarose gel electrophoretogram of RT-PCR products is shown, also including R-actin mRNA, for tap water alone (lane 1), EAW alone (lane 2), aspirin-HCl (lane 3), and EAW plus aspirin-HCl (lane 4). Reprinted from Naito et al. (52), “Chronic Administration with Electrolyzed Alkaline Water Inhibits Aspirin-Induced Gastric Mucosal Injury in Rats through the Inhibition of Tumor Necrosis Factor-α Expression”, © The Editorial Secretariat of JCBN, under the Creative Commons Attribution-NonCommercial-NoDerivatives License CC BY-NC-ND 4.0.

Recent experimental and preclinical evidence suggests that alkaline reduced water may inhibit pro inflammatory mediators, reverse gut microbiota imbalance, restore SCFAs, and enhance antioxidant enzyme activity, leading to histological healing and symptomatic improvement. In an animal trial involving rats with aspirin-hydrochloric acid induced gastric injury, 2 weeks of alkaline reduced water treatment inhibited the increase in tumor necrosis factor alpha messenger RNA expression when compared with tap water (52). This is illustrated through gel electrophoresis results of reverse transcription polymerase chain reaction (RT-PCR) reaction products, in Figure 4.

In the same study, alkaline reduced water significantly inhibited DNA fragmentation, indicating reduced apoptosis in gastric epithelial cells, and also decreased the total area of gastric erosions compared with tap water and sodium hydroxide alkaline solution, which showed no reduction in erosion area (52). The increase in myeloperoxidase levels in the gastric mucosa was also significantly suppressed, suggesting reduced neutrophil accumulation and lower reactive oxygen species production, thereby protecting against gastric mucosal injury and reducing progression to peptic ulcer disease (52).

Peer-reviewed preclinical evidence suggests that hydrogen-rich water may exert anti-inflammatory effects in experimental inflammatory bowel disease models. In a mouse model, hydrogen-rich water was reported to reduce intestinal inflammation by inhibiting endoplasmic reticulum stress and promoting heme oxygenase-1 expression (60). Furthermore, a study investigating alkaline reduced water in mice with ulcerative colitis found that it improved histopathological colonic injury by delaying mucosal damage, inflammation, and edema. Similar to the pattern observed in gastric injury, alkaline reduced water also significantly reduced serum levels of tumor necrosis factor alpha and interleukin 6 in mice with ulcerative colitis (61).

Another study evaluating alkaline reduced water in rat models of inflammatory bowel disease emphasized the role of molecular hydrogen as a scavenger of cytotoxic ROS and free radicals. In that study, elevated plasma ROS levels were significantly suppressed by alkaline reduced water administration at 2 and 4 days after colitis induction in mice. Superoxide dismutase assays also demonstrated a statistically significant increase in enzyme activity, suggesting restoration of antioxidant defense after suppression in inflamed colonic tissue, with subsequent improvement in inflammatory abdominal pain in inflammatory bowel disease rats (62).

### 3.6 Restoration of gut microbiota and antioxidant activity

**Figure 5:**
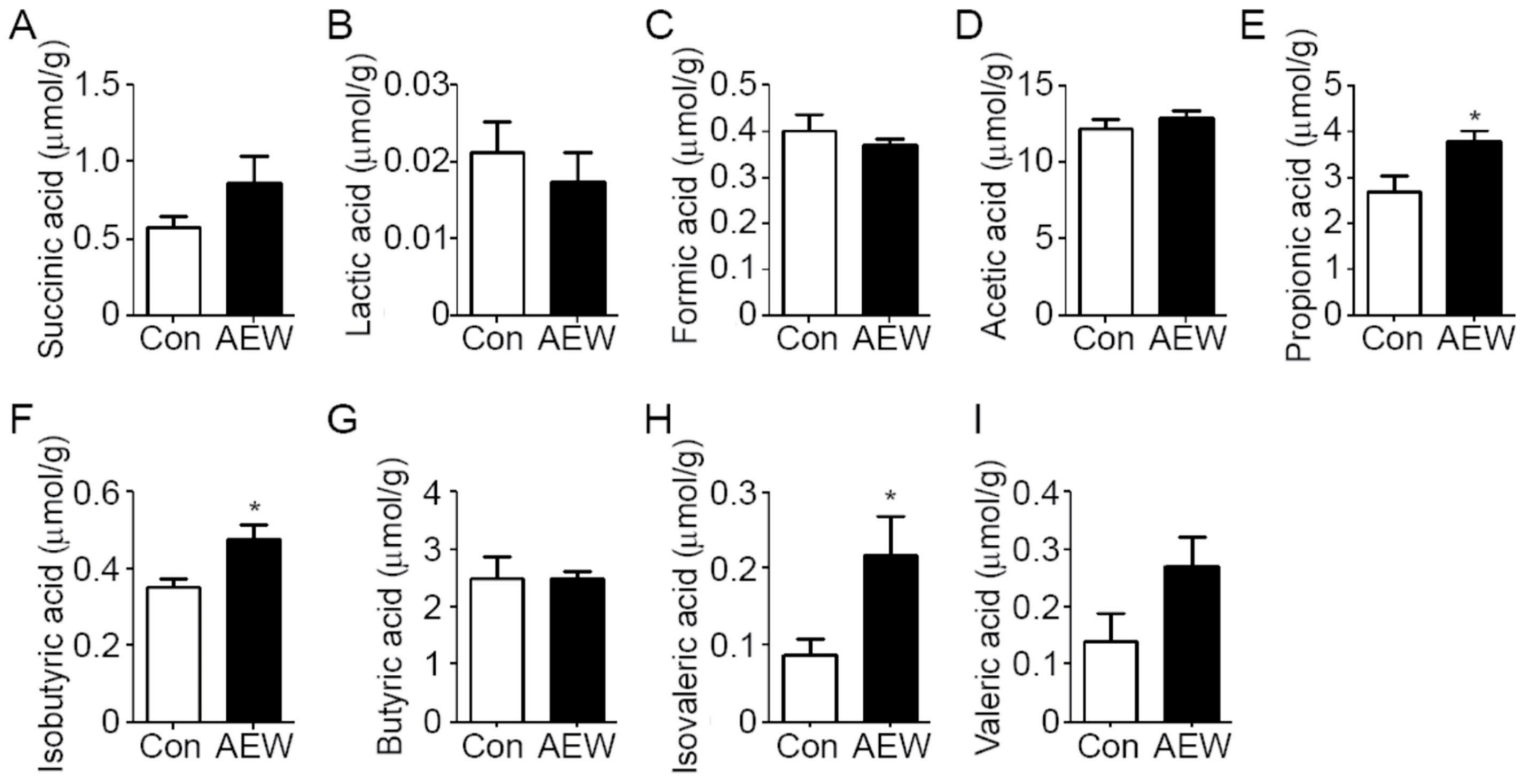
Effects of AEW on short-chain fatty acids in mouse cecal contents. The concentrations of succinic **(A)**, lactic **(B)**, formic **(C)**, acetic **(D)**, propionic **(E)**, isobutyric **(F)**, butyric **(G)**, isovaleric **(H)**, and valeric acid **(I)** in the cecum are shown. The data represent the mean ± SE of seven mice. *p < 0.05, vs. control group (Con) (Mann–Whitney U test). AEW, Alkaline electrolyzed water group. Reprinted from Higashimura et al. (63), “Effects of Molecular Hydrogen-Dissolved Alkaline Electrolyzed Water on Intestinal Environment in Mice”, Copyright © 2018 Medical Gas Research | Published by Wolters Kluwer - Medknow, under the terms of the Creative Commons Attribution-NonCommercial-ShareAlike 4.0 International License (CC BY-NC-SA 4.0).

A recent Japanese study investigating the effects of alkaline reduced water in healthy mice provided important data regarding gut microbiota and SCFA levels. Using 16S ribosomal RNA sequencing, the study found a strong association between 4 weeks of alkaline reduced water administration and increased abundance of *Parabacteroides*, *Rikenellaceae*, *Mucispirillum*, *Butyricimonas*, *Allobaculum*, and *Prevotella* compared with control mice given normal water (63). In parallel, mice receiving alkaline reduced water had significantly higher concentrations of cecal short chain fatty acids such as propionic acid, isobutyric acid, and isovaleric acid, all of which possess anti-inflammatory and antioxidative properties and are associated with increased superoxide dismutase and catalase activity (64). The varying effects of ARW on the proportions of different SCFAs in cecal contents of mice are shown in Figure 5.

Additionally, another paper highlights that, across several studies, alkaline reduced water (ARW) may help prevent intestinal dysbiosis, increase the abundance and diversity of intestinal flora, and reduce oxidative stress (64). Moreover, a study examining the effects of ARW on fecal microbiota and gut integrity reported significantly higher levels of butyric acid in ARW treated mice, together with improvements in intestinal barrier integrity and tight junction integrity in the ileum (65).

## 4 Effects of alkaline water on other organ systems

### 4.1 Role of alkaline water on exercise and bone health

During competition, combat sport athletes experience substantial sweating and an increase in core temperature, both of which can impair muscle strength and reduce motor cortex activation, peripheral stimulation, reaction speed, and power output. Anaerobic exercise also produces lactic acid within the musculoskeletal system. A portion of this lactic acid enters the bloodstream, lowering body pH and disturbing the body’s natural acid base balance. In addition, hydrogen ions are released from muscles in excess of lactate following intense exercise. Alkaline water has therefore been marketed as a nutritional aid because of its claimed antioxidant, acid lowering, and anti-aging properties. Water pH, together with the proportions of sulfate (SO₄^2−^) and bicarbonate (HCO₃^−^), has been proposed to influence hydration status and other therapeutic effects (66–68).

Chycki et al. suggested that alkaline water may produce effects similar to sodium bicarbonate in preventing exercise induced metabolic acidosis. Their study found that the group consuming alkaline water demonstrated statistically significant increases in both average and peak power in the upper and lower limbs. They also showed a significant increase in total work in both limb groups. In addition, the study reported several physiological changes in the alkaline water group, including lower resting lactate concentrations and higher post exercise lactate concentrations. Resting blood pH and both resting and post exercise bicarbonate levels were also increased. Furthermore, post exercise potassium levels and urine pH increased, while urine specific gravity decreased (68).

The study concluded that alkaline water consumption may support athletes by improving hydration status and counteracting exercise induced metabolic acidosis. It also suggested that these effects may enhance the body’s natural buffering system and potentially improve exercise performance. Improved anaerobic capacity in athletes consuming alkaline water was proposed to result from changes in blood pH and bicarbonate levels, which may facilitate the removal of lactate and hydrogen ions from muscle. In addition, the study suggested that alkaline water improved hydration and reduced blood viscosity, which may help transport metabolic byproducts and thereby support athletic performance. However, this protocol involved trained athletes consuming approximately 3–4 L/day of alkaline water under exercise conditions and should not be generalized to routine clinical use. Large-volume water intake may increase the risk of dilutional hyponatremia, particularly in individuals with chronic kidney disease, heart failure, impaired electrolyte regulation, or other medical vulnerabilities (68).

With respect to bone health, osteoporosis is characterized by reduced bone density and the loss of bone microarchitecture, leading to increased fragility and a higher risk of fractures. In menopausal women, this occurs because of physiological and biochemical changes, increased bone turnover, and increased bone resorption. An increase in pH has been associated with a reduction in the number of osteoclasts, which are responsible for bone resorption, and an increase in osteoblast activity, which is responsible for bone formation (69). Excess acidity can be problematic, and the body compensates through buffering systems involving bone minerals, carbon dioxide expiration, and renal acid excretion to maintain normal pH, whereas aging reduces renal acid excretion (70).

In relation to dietary acidity, one study reported that acid producing diets increase bone resorption, whereas alkaline or bicarbonate rich diets reduce it. Acidic diets may promote the release of calcium, largely stored in the body as phosphate or carbonate salts, in order to help maintain blood pH. As a result, urinary calcium excretion may reach approximately 480 g, representing nearly half of total skeletal calcium. Moreover, chronic metabolic acidosis in rats has been shown to reduce total bone mineral density. Modifying an acidogenic diet therefore appears to have important implications for bone health (69).

Fasihi et al. investigated the effect of alkaline water on bone mineral density in postmenopausal women; however, changes in spine and femur T-scores were not statistically significant compared with the control group. Therefore, the findings should not be presented as clear evidence that alkaline water improves bone density, but rather as preliminary evidence requiring longer follow-up and further controlled investigation (69).

A study conducted by Emma Wynn found that urinary pH and bicarbonate excretion increased in individuals who consumed mineral water, while parathyroid hormone (PTH) and serum C terminal telopeptide (S CTX) levels decreased (70). Calcium rich mineral water was shown to influence short term trials, particularly under conditions of calcium and estrogen deficiency, where a reduction in bone resorption was observed. In healthy individuals, supplementation with potassium bicarbonate, potassium citrate, or bicarbonate rich mineral water reduced calciuria and markers of bone resorption. This study further indicates that calcium rich mineral water may reduce bone resorption in postmenopausal women with calcium deficiency. These findings suggest that alkaline water rich in bicarbonate may inhibit bone resorption, even in the setting of a free diet and high calcium intake (70).

In premenopausal women consuming a balanced and calcium sufficient diet, both bicarbonate rich and calcium rich mineral water were more effective in reducing bone resorption than calcium rich mineral water alone. Nevertheless, further research is needed to determine whether these beneficial effects are maintained over the long term and whether they ultimately translate into increased bone mineral density (70).

### 4.2 Alkaline water anti-aging effects and skin care

Telomere length and telomerase activity are involved in the molecular mechanisms of aging. Telomeres are located at the ends of DNA strands and protect them from recombination, degradation, and interchromosomal fusion. However, when telomeres shorten, they trigger a DNA damage response that results in cell death or cellular senescence. Studies suggest that the accumulation of oxidants accelerates the aging process. Acidic environments, such as those observed in malignant tumors and type 2 diabetes, may also influence telomere length, which is an important marker of cellular aging. In addition, a slightly acidic environment at pH 6.8 can activate human telomerase to selectively lengthen short telomeres, which are associated with reduced cell viability and aging. Logozzi et al. proposed a relationship between alkaline water and the molecular aging process (71).

**Figure 6:**
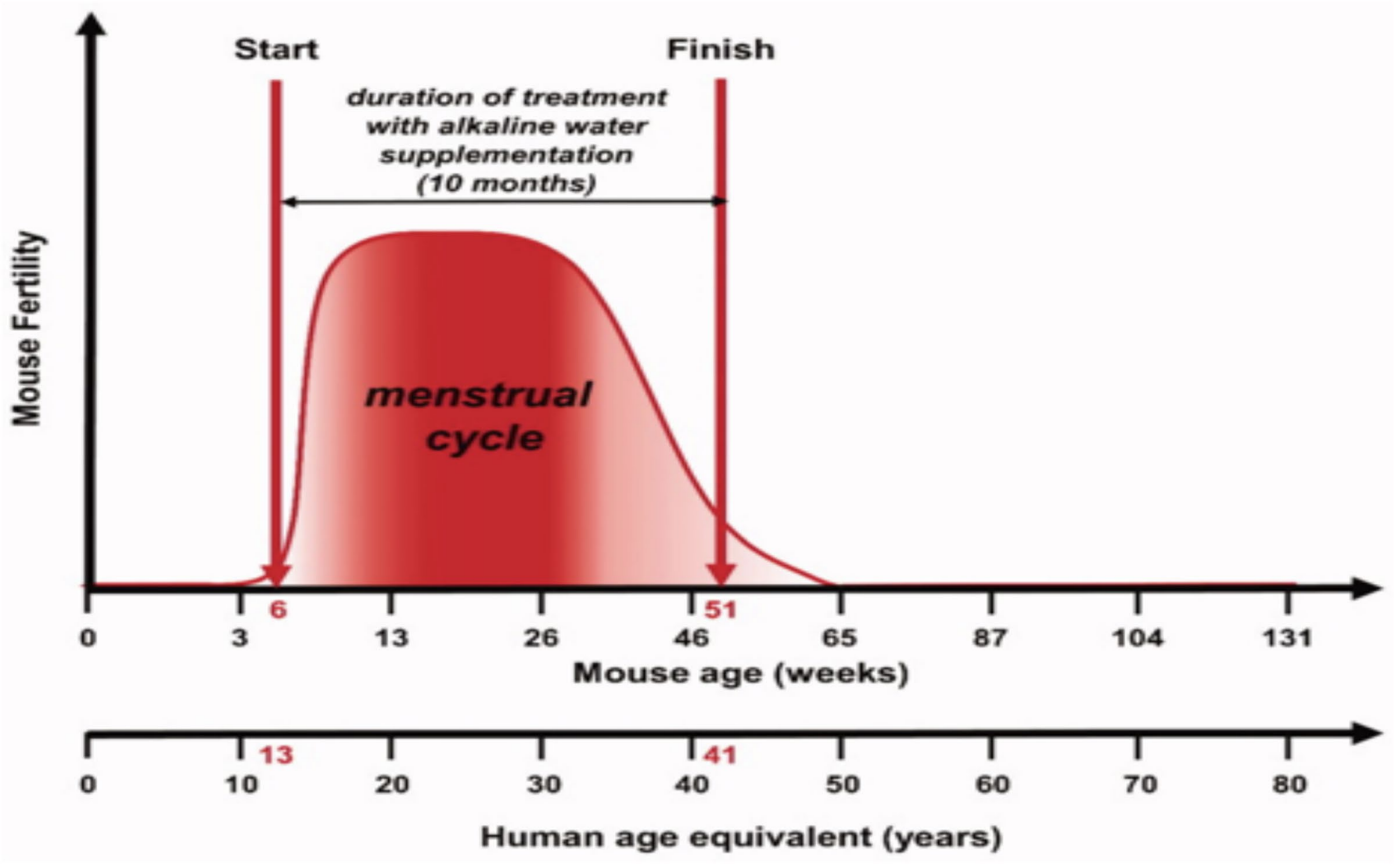
Experimental design illustrating the evaluation of alkaline water administration (pH 9.0) in C57BL/6 J female mice. Treatment was conducted for 10 months, from 6 weeks of mice age until 51 weeks of mice age which corresponds to (13–41 years old) of human women age. Reprinted from Logozzi et al. (71), “In Vivo Antiaging Effects of Alkaline Water Supplementation,” published in Journal of Enzyme Inhibition and Medicinal Chemistry by Taylor & Francis, under the terms of the Creative Commons Attribution 4.0 International License (CC BY 4.0).

They supported this by conducting an *in vivo* experiment that demonstrated reduced levels of reactive oxygen species (ROS), increased superoxide dismutase 1 (SOD 1) and glutathione (GSH) levels, greater telomerase activity and telomere length, and an increased number of ovarian germ cells (71). These findings suggest that daily intake of alkaline water may exert anti-aging effects. The authors also proposed that alkaline water stimulates powerful antioxidant enzymes such as SOD, which counter the production and accumulation of free radicals throughout the body. It has therefore been suggested that consuming alkaline water containing essential elements may contribute to slowing or improving aspects of the cellular aging process. Moreover, the observed increase in ovarian germ cells suggests a possible effect on prolonged fertility. In this study, they treated mice starting from 6 weeks of age until 51 weeks of age (which corresponds to the fertile window of a human woman from approximately 13–41 years old) for a period of 10 months with AWS as shown in Figure 6 (71).

Oxidants also affect the skin’s acid mantle, which normally ranges from pH 4.5–5.5, although broader estimates place it between pH 4.0 and 7.0 (72, 73). This acid mantle helps maintain microbiome homeostasis, inhibits pathogen growth, and regulates enzymatic activity related to barrier function. Because the skin is in direct contact with the external environment, it is exposed to multiple oxidant sources. The antioxidant activity of alkaline water may therefore help prevent aging processes and certain dermatoses. In addition, its antioxidant properties may help counter the effects of ROS, including skin aging, collagen degradation, and inflammatory dermatoses such as psoriasis and eczema. Some preliminary studies have indicated positive effects of alkaline water on skin hydration, barrier repair, and inflammation control. However, clinical evidence remains limited. Different skin pH ranges affect the health of skin condition and the barrier integrity, all of which is clearly demonstrated in Table 2 (73).

**Table 2:** The table demonstrates the relationship between skin pH and the health of the skin (73).

Skin pH range	Skin condition	Barrier integrity	Remarks
4.5–5.5	Healthy skin	Optimal	Balanced microbiome, intact barrier
5.6–6.5	Mild imbalance	Slightly impaired	Increased bacterial activity (e.g., acne)
6.6–7.5	Barrier disruption	Compromised	TEWL, dryness, irritation
>7.5	Chronic alkalinity	Severely compromised	Inflammation, prone to dermatitis

Reprinted from Khandelwal (73), “Alkaline Water in Skin Care: A Mini Review of Dermatological Benefits and Mechanisms,” published in Journal of Drug Delivery and Biotherapeutics by Sennos Biotech Pvt. Ltd., under the terms of the Creative Commons Attribution 4.0 International License (CC BY 4.0).

Khandelwal (73) reported that alkaline water may help regulate variations in skin pH, provide antioxidant effects, improve hydration, support detoxification processes, improved skin tone and inflammation, and skin barrier repair. These effects of alkaline water in skincare depend on its concentration, source, and route of administration, all of which is shown in Table 3 (73).

**Table 3:** Alkaline water effects through anti-oxidation on human skin (73).

Model/system used	Route of use	Observed dermatological benefit
Human subjects	Oral intake	Improved hydration, decreased oxidative markers
Topical mist (cosmetic)	Topical use	Reduced redness, enhanced skin tone
UV-damaged animal skin	Oral + topical	Prevention of collagen degradation
Atopic dermatitis model	Topical therapy	Inflammation control, barrier repair

Reprinted from Khandelwal (73), “Alkaline Water in Skin Care: A Mini Review of Dermatological Benefits and Mechanisms,” published in Journal of Drug Delivery and Biotherapeutics by Sennos Biotech Pvt. Ltd., under the terms of the Creative Commons Attribution 4.0 International License (CC BY 4.0).

Although its antioxidant properties have been demonstrated in laboratory settings, achieving the same benefits on human skin remains difficult because of issues related to skin penetration and bioavailability. For this reason, the study suggested that alkaline water should be considered a complementary component of a skincare routine rather than a stand-alone treatment. To address bioavailability limitations, nanotechnology and cosmeceuticals may improve alkaline water delivery, enabling targeted application and more sustained antioxidant effects. The study also suggested that combining alkaline water with other components such as hyaluronic acid or botanical extracts may produce synergistic improvements in skin hydration, elasticity, and overall appearance. In addition, the use of alkaline water in post procedural care, such as after laser treatment or chemical peeling, may support faster healing and reduced inflammation (73).

### 4.3 Alkaline water effect on type 2 diabetes mellitus

Hyperglycemia is a defining feature of type 2 diabetes mellitus (T2DM) and is closely associated with oxidative stress and inflammatory dysregulation. Ma et al. investigated the effects of alkaline reduced water (ARW) with a pH of 8.5 in a C57BL/6 mouse model of T2DM induced by a high fat diet and streptozotocin. After diabetes induction, mice were treated for 4 weeks with tap water, metformin, or ARW. The authors assessed fasting blood glucose, oral glucose tolerance, white blood cell profiles, inflammatory markers, and oxidative stress markers, including reactive oxygen species, nitric oxide, glutathione peroxidase, and catalase. ARW treatment was reported to enhance antioxidant defenses, moderate inflammation, improve cellular metabolism, and reduce hyperglycemia related adverse effects in this animal model. However, because this evidence comes from a preclinical mouse study rather than a human clinical trial, these findings should be interpreted cautiously and should not be used to support direct clinical recommendations for patients with T2DM (74).

### 4.4 Alkaline water effect on dental enamel and oral microbiota

A recent *in vitro* double-blind experimental study found that human teeth exposed to alkaline reduced water for 7 and 15 days, showed a statistically significant reduction in salivary bacterial content such as *Streptococcus salivarius, Staphylococcus aureus,* and *Lactobacillus casei* compared to a normal water control (75). Meanwhile, no significant difference in bacterial content was observed after only 3 days of exposure. Moreover, the study suggests that alkaline water can stimulate saliva production and can prevent acidic conditions in the oral cavity, thus preventing enamel demineralization. With that in mind, the study also highlighted the importance of Alkaline water in maintaining oral hygiene when used as a daily mouthwash, particularly in individuals wearing orthodontic apparatus or with physical and mental challenges, who may have more difficulty in maintaining oral hygiene. However, further *in vivo* trials with larger sample sizes are required to determine the oral antimicrobial capacity of alkaline water (75).

## 5 Risks and cautions

### 5.1 Disruption of acid–base homeostasis


$${\mathrm{CO}}_{2}+H_{2}O⇌H_{2}{\mathrm{CO}}_{3}⇌H^{+}+{{\mathrm{HCO}}_{3}}^{-}.$$

A primary concern is the disruption of normal acid base balance. The body’s buffering systems, including renal, respiratory, and plasma protein mechanisms, maintain arterial pH within a narrow physiological range. Chronic ingestion of highly alkaline water may induce metabolic alkalosis, which is characterized by increased serum bicarbonate, hypoventilation, and compensatory renal acid excretion (76). Mild metabolic alkalosis may be asymptomatic or may present with nausea, paraesthesia, or cognitive disturbances, whereas severe alkalosis can lead to cardiac arrhythmias, neuromuscular irritability, and impaired oxygen delivery due to a leftward shift of the oxyhemoglobin dissociation curve.

Although healthy kidneys usually help maintain acid base homeostasis by regulating bicarbonate reclamation, acid excretion, and ammonium handling, this compensatory capacity may be impaired when renal function is reduced (77). Therefore, individuals with renal insufficiency or other conditions that limit normal acid base compensation may be more vulnerable to clinically significant alkalosis when exposed to excessive alkali loads. In addition, infants and young children, whose renal regulatory capacity is still developing, may be more susceptible to disturbances in pH and electrolyte balance. For these populations, routine consumption of high pH water should be approached with caution and considered within a medical context.

### 5.2 Impact on gastrointestinal physiology

Although alkaline water may temporarily inactivate pepsin and reduce laryngopharyngeal reflux (2), chronic intake may interfere with normal digestive physiology. Gastric acidity is essential for protein denaturation, nutrient absorption, and defense against ingested pathogens. Persistent neutralization of gastric acid by high pH water may impair protein digestion and reduce the absorption of important micronutrients such as iron, calcium, magnesium, and vitamin B12. These effects may be more relevant in individuals with hypochlorhydria or in those receiving proton pump inhibitor (PPI) therapy, because long-term acid suppression has been associated with potential adverse effects including nutrient deficiencies, particularly vitamin B12 and magnesium, and increased susceptibility to enteric infections (78). Therefore, additional chronic intake of high-pH water should be interpreted cautiously in patients who already have reduced gastric acidity or are receiving long-term acid-suppressive therapy.

Electrolytically produced alkaline water may also contain dissolved molecular hydrogen and other electrolysis-related physicochemical changes, making its gastrointestinal effects difficult to attribute to pH alone. In a human study, daily ingestion of alkaline electrolyzed water containing hydrogen was associated with changes in self-reported gastrointestinal symptoms, but the available data remain limited and do not establish long-term gastrointestinal safety with chronic high-pH water intake (79). Therefore, although adverse gastrointestinal effects appear uncommon, moderation and individualized assessment remain important when high-pH or electrolyzed water is used as part of daily hydration.

### 5.3 Effects on renal function and electrolyte homeostasis

The kidneys play a central role in regulating acid base balance and electrolyte homeostasis. Habitual intake of mineral enriched alkaline water may increase the renal solute load, thereby raising the risk of electrolyte disturbances such as hyperkalemia, hypercalcemia, or metabolic alkalosis. This concern is particularly relevant in individuals with chronic kidney disease (CKD), whose reduced renal excretory function limits the elimination of excess alkali and minerals.

Magnesium-rich alkaline waters, often marketed for enhanced mineralization, may increase magnesium exposure, which is most clinically relevant in individuals with impaired renal function because the kidneys are the main route for magnesium excretion. In patients with reduced renal clearance, excessive magnesium intake may contribute to clinically significant hypermagnesemia, with manifestations ranging from mild gastrointestinal or neuromuscular symptoms to hypotension, bradycardia, respiratory depression, and cardiopulmonary arrest at higher serum magnesium levels, as summarized in Table 4. Similarly, alkaline waters with high sodium concentrations may worsen edema or hypertension in patients with heart failure who are receiving diuretic therapy (80).

**Table 4:** Clinical manifestations associated with increasing serum magnesium levels, adapted from Tinawi (80).

Serum Mg levels	Clinical manifestations
2.7–5.0 mg/dL (1.1–2.0 mmoL/L)	Usually no symptoms
5.1–7.0 mg/dL (2.1–2.9 mmoL/L)	Mild symptoms: nausea, vomiting, dizziness, weakness, drowsiness, diminished deep tendon reflexes.
7.1–12 mg/dL (3.0–5.0 mmoL/L)	Urinary retention, lethargy, ileus, flushing, sleepiness, blurred vision, confusion, loss of deep tendon reflexes
>12 mg/dL (> 5.0 mmoL/L)	Flaccid paralysis, respiratory depression, apnea, low BP, bradycardia, complete heart block. Symptoms can progress to coma and cardiopulmonary arrest

Adapted from Tinawi (80), “Disorders of Magnesium Metabolism: Hypomagnesemia and Hypermagnesemia,” published in Archives of Clinical and Biomedical Research by Fortune Journals, under the terms of the Creative Commons Attribution 4.0 International License (CC BY 4.0).

More directly relevant clinical evidence comes from a pediatric nephrology case report by Kermond et al., in which alkaline water was implicated in calcium-alkali syndrome in a child with chronic kidney disease who was also receiving calcium carbonate therapy. The patient developed the characteristic triad of hypercalcemia, metabolic alkalosis, and acute-on-chronic kidney injury, with improvement in creatinine, calcium, and acid base status after alkaline water was discontinued. This case does not establish that alkaline water is directly nephrotoxic in healthy individuals, but it highlights that absorbable alkali and mineral content may become clinically significant in vulnerable patients, especially those with reduced glomerular filtration rate, impaired renal excretion, or concurrent calcium-based medications. Therefore, the main renal concern is not proven direct tubular toxicity from alkaline or electrolyzed water itself, but the possibility that chronic or excessive intake of high-pH or mineral-rich water may worsen alkali load, electrolyte imbalance, fluid status, or acid base interpretation in susceptible patients. As a result, the balance between potential benefits from mineral intake and the risk of renal overload requires individualized evaluation, especially in populations with compromised renal or cardiovascular function (81).

### 5.4 Risks associated with microbiological and chemical contaminants

The safety profile of alkaline water is strongly influenced by the method used to produce it. Commercial bottled alkaline water is generally manufactured through controlled mineral supplementation, whereas domestic ionizers raise pH through electrolysis. Unlike bottled products, home ionizing devices may vary in design, electrode composition, maintenance quality, and output chemistry, which can affect the consistency and safety of the final water product (82). Electrolyzer electrodes are commonly composed of titanium with platinum coating, but device quality and manufacturing standards may influence the potential for electrode-derived metal exposure or other impurities (82). Although clinically significant toxicity from regulated devices has not been established, chronic exposure to poorly controlled contaminants may be more relevant in vulnerable populations such as children, pregnant women, and individuals with impaired renal clearance (82).

In addition, inadequate maintenance of ionizer filters and reservoirs may compromise water safety. Stagnant water and poor disinfection practices can promote microbial growth or biofilm formation within tubing and storage compartments. Consumption of contaminated water may therefore lead to gastrointestinal infections or dysbiosis, particularly when devices are not cleaned according to manufacturer guidance or when filters are used beyond their intended lifespan (17, 18, 82).

In view of these concerns, strict quality control and consumer education are essential. Users should confirm that devices meet recognized safety standards and should follow rigorous cleaning and filter replacement practices. When product quality cannot be reliably ensured, commercially bottled, mineral-balanced alkaline water may represent a more standardized alternative, although it should still be evaluated according to labeled mineral composition, pH, storage conditions, and regulatory compliance (82).

### 5.5 Overconsumption and water intoxication

Like any other type of water, alkaline water can contribute to water intoxication when consumed in excessive amounts. Overhydration can dilute plasma sodium concentration, resulting in hyponatremia, a potentially life-threatening electrolyte disturbance characterized by confusion, seizures, and cerebral edema. Endurance athletes may be especially vulnerable because they may attempt to replace sweat losses by drinking large volumes of low electrolyte alkaline water instead of isotonic fluids formulated for proper rehydration (83).

Acute hyponatremia develops when water intake exceeds the kidney’s excretory capacity, causing rapid changes in intracellular osmolarity. The resulting osmotic imbalance leads to cerebral cell swelling, increased intracranial pressure, and neurological manifestations ranging from headache to coma. Even mild reductions in serum sodium can impair cognitive function and neuromuscular coordination, thereby increasing the risk of accidents or heat related illness during physical exertion. Preventive measures include adjusting fluid intake according to thirst and alternating alkaline water with electrolyte balanced solutions during prolonged exercise or heat exposure. Individuals who are following medically supervised hydration plans, such as those with heart failure or renal dysfunction, should seek clinical advice before introducing large volumes of alkaline water into their routine (83).

### 5.6 Interference with medications and nutrient absorption

Chronic consumption of alkaline water, which increases gastric pH, may influence the pharmacokinetics of many orally administered medications. Several orally administered medications have pH-dependent solubility or absorption and may be affected by factors that raise gastric pH. Examples include azole antifungals such as ketoconazole and itraconazole, certain antiretroviral agents, and iron supplements such as ferrous sulfate (84). Under alkaline conditions, these agents may show reduced bioavailability, potentially lowering therapeutic efficacy and increasing the risk of treatment failure.

Altered gastric acidity may also impair the absorption of important micronutrients. Non-heme iron, calcium, magnesium, and vitamin B12 depend on acidic conditions for solubilization or absorption-related processes. Long-term acid suppression, particularly with proton pump inhibitor (PPI) therapy, has been associated with potential micronutrient deficiencies, including vitamin B12 and magnesium deficiency, while altered gastric pH may also influence the dissolution and absorption of selected orally administered drugs (78, 84). Therefore, chronic intake of high-pH alkaline water should be interpreted cautiously in patients with pre-existing deficiencies, malabsorption syndromes, or long-term acid-suppressive therapy.

Drug-nutrient interactions may further intensify these effects. Concomitant use of proton pump inhibitors (PPIs) or H2 receptor antagonists, both of which increase gastric pH, may alter the bioavailability of pH-sensitive medications. Healthcare providers should therefore consider hydration habits during medication reviews and advise patients on appropriate timing of water consumption in relation to drug administration (78, 84).

Taken together, these observations emphasize the importance of considering the biochemical environment in which medications and nutrients are processed. Although occasional consumption of mildly alkaline water is unlikely to produce clinically significant interference, chronic or high-volume intake may alter gastric physiology sufficiently to justify pharmacological caution. Patient education regarding timing of fluid intake, adherence to prescribed therapy, and appropriate hydration choices represents an important yet often overlooked aspect of safe treatment management (84).

### 5.7 Dermatological and musculoskeletal considerations

Alkaline water is often promoted for its supposed benefits in skin and musculoskeletal health. However, excessive alkalinity may disrupt the skin’s natural acid mantle, a slightly acidic protective barrier that helps prevent microbial invasion and limits transepidermal water loss. Disruption of this barrier may trigger or worsen conditions such as xerosis, eczema, and irritant dermatitis. For this reason, individuals with pre-existing skin disorders or impaired skin integrity should avoid prolonged exposure to topical applications or water with markedly elevated pH (72, 73).

Concerning the musculoskeletal system, the evidence remains inconclusive. Some studies suggest that bicarbonate rich mineral waters may reduce bone resorption by neutralizing dietary acid loads, whereas sodium dominant formulations may theoretically offset these benefits by increasing urinary calcium loss. This distinction is important because the skeletal relevance of alkaline water appears to depend more on mineral composition than on pH alone. Park et al. evaluated the relationship between sodium intake, urinary calcium excretion, and bone resorption in postmenopausal women with low bone mass. They reported that 24-h urinary calcium excretion was significantly higher in the high sodium intake group when sodium exposure was assessed using 24-h urinary sodium collection, although this difference was not significant when sodium intake was estimated using a food frequency questionnaire (85). These findings are summarized in Table 5 (85).

**Table 5:** Urinary calcium excretion and serum C-terminal telopeptide of type I collagen according to low and high sodium intake groups, adapted from Park et al. (85).

Sodium intake grouping	Outcome assessed	Main finding
Low sodium intake group (<2 g/day) versus high sodium intake group (≥2 g/day), based on food frequency questionnaire	24-h urinary calcium excretion	No significant difference between groups
Low sodium intake group (<2 g/day) versus high sodium intake group (≥2 g/day), based on 24-h urinary sodium collection	24-h urinary calcium excretion	Significantly higher urinary calcium excretion in the high sodium group
Low sodium intake group (<2 g/day) versus high sodium intake group (≥2 g/day), based on 24-h urinary sodium collection	Serum C-terminal telopeptide of type I collagen (CTX-I)	Higher tendency in the high sodium group, but not statistically significant

Adapted from Park et al. (85), Copyright © 2014 The Korean Society for Bone and Mineral Research, licensed under the terms of the Creative Commons Attribution-NonCommercial 3.0 Unported License (CC BY-NC 3.0).

These findings suggest that sodium load may be an important confounding factor when interpreting claims that alkaline water supports bone health. Sodium-rich alkaline water should therefore not be assumed to have the same skeletal implications as bicarbonate-rich mineral water containing balanced calcium and magnesium. Although current evidence does not establish that alkaline water causes clinically significant bone mineral loss, products with high sodium content may contribute to calciuria and should be interpreted cautiously, especially in individuals at risk of osteoporosis or negative calcium balance. Further randomized controlled trials using standardized water compositions are needed to clarify dose response relationships and assess long term skeletal safety (85).

### 5.8 Vulnerable populations

Certain demographic and clinical groups are more susceptible to imbalances associated with alkaline water consumption. Patients with chronic kidney disease or renal tubular dysfunction have reduced capacity to regulate acid base balance and are therefore particularly vulnerable to metabolic alkalosis, electrolyte disturbances, and mineral accumulation. This caution is also relevant to individuals with pre-existing acid base abnormalities, recurrent metabolic alkalosis, chronic respiratory disease with renal compensation, prolonged vomiting, gastric fluid loss, or conditions requiring loop or thiazide diuretics. In these settings, renal bicarbonate handling, acid excretion, and compensatory mechanisms may already be impaired or actively engaged, meaning that chronic or excessive intake of high pH or mineral rich alkaline water could worsen alkalemic tendencies or complicate clinical acid base management (76, 77, 86).

Individuals using acid suppressive medications such as proton pump inhibitors (PPIs) or H2 receptor antagonists also require caution. These medications increase gastric pH and, particularly with long-term proton pump inhibitor therapy, have been associated with potential adverse effects including impaired micronutrient status, especially vitamin B12 and magnesium, and increased susceptibility to enteric infections (78). Additional chronic intake of high pH alkaline water may further influence gastric acidity and should therefore be interpreted cautiously in patients with pre-existing deficiencies or long-term acid suppression therapy. Infants and young children, whose renal and gastrointestinal systems are still developing, may also be at greater risk of electrolyte disturbances and impaired nutrient absorption (83).

Pregnant individuals represent another important group requiring specific caution. Pregnancy is associated with physiological changes in acid base balance, including mild respiratory alkalosis with renal bicarbonate compensation, as well as changes in renal filtration, sodium balance, and calcium metabolism (87). Because pregnancy also increases nutritional demands and may alter electrolyte handling, sustained intake of high pH or heavily mineralized alkaline water should not be assumed to be universally safe. Although occasional consumption of mildly alkaline water is unlikely to be harmful in healthy pregnancies, chronic or excessive intake may theoretically complicate acid base interpretation, electrolyte balance, or micronutrient status, particularly in individuals with vomiting, renal disease, hypertensive disorders of pregnancy, long-term acid suppression therapy, or pre-existing nutritional deficiencies (78, 87).

Patients with cardiac disease receiving loop or thiazide diuretics may be more prone to electrolyte depletion, while athletes consuming large volumes of low electrolyte alkaline water during endurance exercise may face an increased risk of dilutional hyponatremia. Exercise associated hyponatremia has been reported in approximately 5 to 15 percent of endurance athletes, with severe cases occurring when excessive hypotonic fluid intake overwhelms the kidneys’ ability to excrete water (83).

These groups would benefit from individualized hydration advice under clinical supervision. Although moderate consumption of mildly alkaline water is generally considered safe for healthy adults, chronic or excessive intake, particularly from unregulated ionization devices, should be discouraged in vulnerable populations. Public health messaging should prioritize evidence-based guidance and actively counter misconceptions that portray alkaline water as universally beneficial (8, 86).

## 6 Metabolism and immunology

### 6.1 Metabolic effects

This section examines claims regarding the benefits of alkaline water in metabolism and immune system modulation. By evaluating both human and animal studies, with particular focus on lipid profiles, glucose regulation, and redox related immunological responses, it aims to determine whether biological trends support these claims. Across the reviewed studies, the primary parameters assessed included lipid profiles, specifically low density lipoprotein cholesterol (LDL C), high density lipoprotein cholesterol (HDL C), triglycerides (TG), and total cholesterol (TC), all measured in mg/dL; glucose homeostasis, evaluated using fasting plasma glucose, oral glucose tolerance tests (OGTTs), insulin levels, and homeostatic model assessment of insulin resistance (HOMA IR) indices; and weight related measures including body weight, body mass index (BMI), waist circumference, body fat percentage, and occasionally lean or fat free mass (88–93). In addition to these main parameters, several studies also investigated acid–base balance using urinary pH, renal net acid excretion, and blood pH measurements (94, 95).

Regarding glucose profiles, the available human evidence remains limited and inconsistent. In one randomized controlled trial involving individuals with metabolic syndrome, high-concentration hydrogen-rich water was associated with modest improvements in blood glucose, hemoglobin A1c, lipid parameters, and selected inflammatory or redox biomarkers after 24 weeks of consumption (88). In contrast, a randomized cross-over study conducted in healthy young adults found no differential effect of alkaline versus neutral drinking water on glucose regulation after a 2-week intervention (89). In animal studies using diabetic rodent models, electrolyzed reduced water was associated with more favorable outcomes, including reduced blood glucose and improved glucose tolerance (92). However, unlike the animal findings, these trends were not consistently reproduced in human studies (88, 89).

In the lipid domain, one randomized controlled trial using high-concentration hydrogen-rich water reported modest improvements in cholesterol and glucose-related parameters in patients with metabolic syndrome (88), whereas studies using bicarbonate-rich mineral waters have reported mixed findings depending on population, water composition, and study design (90, 91). Results for high density lipoprotein cholesterol were inconsistent. One study reported a slight increase after several weeks of hydrogen rich or alkaline reduced water intake, whereas another found no statistically reliable difference. Overall, the studies (88–91) demonstrated inconsistent effects on lipid parameters, with limited clinical relevance and insufficient support for claims of meaningful metabolic benefit. This study showed modestly favorable directional trends, suggesting at most a limited potential benefit. Differences in lipid related outcomes likely reflect variations in intervention duration, baseline metabolic status of participants, and water chemistry or composition, and therefore require further investigation before any broader conclusions can be drawn.

In animal studies, interventions involving hydrogen rich water produced more noticeable effects in diseased models than in healthy controls (92, 93). One diabetic rodent study reported improvements in glucose-related outcomes after electrolyzed reduced water intake, while metabolic effects in non-diabetic controls were less pronounced (92). Another animal study demonstrated similar trends, including reductions in low density lipoprotein cholesterol by about 10 percent, total cholesterol by 10–15 percent, and hepatic fat deposition by approximately 30–35 percent on histological assessment (93).

In the same study, high density lipoprotein cholesterol increased by about 5–7 percent and insulin sensitivity improved, with homeostatic model assessment of insulin resistance decreasing by approximately 25 percent. These findings suggest a redox mediated improvement in dyslipidemia under conditions of metabolic stress. Nevertheless, these results remain internally valid only and cannot be directly translated into clinical practice. Physiological differences between rodents and humans, the fact that improvements were observed mainly under experimentally induced metabolic stress, the short duration of the animal studies, and the higher concentrations of mineralized or hydrogen enriched water consumed by rodents all limit their applicability to humans.

Another study evaluating mineral-based alkaline bottled water reported increases in urinary and blood pH, suggesting measurable effects on acid–base markers in healthy adults. However, these findings were based on surrogate physiological markers and do not establish broader claims of systemic metabolic enhancement or clinical benefit (95).

From a biological and physiological perspective, study (95) suggests that mineral-based alkaline bottled water may influence short-term acid–base markers, including urinary and blood pH. However, because these outcomes were surrogate markers in healthy adults, they should not be interpreted as evidence of clinically meaningful metabolic enhancement or disease prevention. Overall, the available research lacks strong clinically meaningful endpoints. The evidence comes from heterogeneous study designs with differing methods and inclusion criteria, and more favorable results were seen mainly in animal models. As a result, the direction of the evidence remains inconsistent and further study is required. A summary of the metabolic outcomes of water intervention in human and animal studies is demonstrated in Table 6.

**Table 6:** Metabolic outcomes of alkaline water interventions in human and animal studies.

Directionality	Population/model	Design	Intervention	Duration	Outcomes	Key findings	References
++	Adults (MetS)	RCT	High-concentration hydrogen-rich water	24 weeks	Glucose, Lipids, Insulin resistance	↓ glucose-related markers, improved lipid profile and inflammatory/redox biomarkers	(88)
0	Healthy Adult Participants	Randomized cross-over intervention	Alkaline versus neutral drinking water	Two 2-week intervention periods	Gut microbiota and glucose regulation	No differential effect on glucose regulation or gut microbiota	(89)
+	Moderately hypercholesterolemic young adults	Clinical intervention	Sodium-bicarbonated mineral water	8 weeks	Lipid profile, glucose, inflammatory/endothelial markers	Improved lipid profile; glucose tended to decrease; triacylglycerol unchanged	(90)
0	Moderately hypercholesterolemic adults	Randomized double-blind cross-over pilot study	High-bicarbonate mineral water versus low-mineral water	8 weeks	Fasting and postprandial lipemia	No significant between-water difference; limited pilot findings	(91)
+++	Diabetic mouse models	Preclinical animal study	Electrolyzed Reduced Water	6 weeks in STZ-induced diabetic mice; 29 days in db/db diabetic mice	Blood glucose, glucose tolerance	↓ blood glucose; improved glucose tolerance	(92)
+++	Diabetic rats	Preclinical Animal Model	Alkaline Reduced Water	~6–8 weeks	Lipids, Insulin Resistance	↓ TG, ↓ LDL, ↓ insulin resistance, ↑ adiponectin	(93)
+	Healthy adults	Controlled intervention study	Mineral-based alkaline bottled water	2-week treatment period within a 4-week protocol	Urinary pH, blood pH, hydration markers	↑ urinary and blood pH; improved hydration markers; surrogate outcomes only	(95)

Description: Directionality of outcomes is represented as follows: +++ indicates strong positive effects, ++ moderate positive effects, + weak or modest effects, and 0 no statistically significant effect. db/db, genetically diabetic db/db mouse model; LDL, low-density lipoprotein; MetS, metabolic syndrome; RCT, randomized controlled trial; STZ, streptozotocin; TG, triglycerides.

### 6.2 Immunological and redox effects

The proposed immunomodulatory effects of alkaline water are thought to occur through inhibition of pro inflammatory signaling and regulation of redox reactions (92–97). However, there is no convincing evidence that these mechanisms improve immune function to a clinically meaningful extent in humans (88, 89, 96). In preclinical and experimental studies, the findings were directionally more consistent. In one diabetic rat study, antioxidant activity increased, with rises in superoxide dismutase, glutathione peroxidase, and catalase, alongside reductions in oxidative stress markers such as malondialdehyde and reactive oxygen species. Similar patterns were observed in other experimental and clinical studies, which reported reductions in oxidative and inflammatory responses, although the measured biomarkers differed between studies (93, 97). One *in vitro* alkaline reduced water study reported reversal of oxidative stress-related immunokine changes, including interleukin 6, interleukin 10, monocyte chemoattractant protein, tumor necrosis factor alpha, and regulated upon activation, normal T cell expressed and secreted (RANTES) (98).

Despite these experimental findings, immune modulation of this magnitude has not been reproduced in human populations (88, 89, 96). Human trial results were inconsistent and variable. Among the available studies, only three provided relevant data on immune modulation. Two studies (88, 96) reported mild improvements in oxidative stress-related biomarkers, although the measured markers differed between studies. One of these involved patients with metabolic syndrome (88), while the other assessed healthy individuals (96). In contrast, another randomized cross-over study in healthy adults found no differential effect of alkaline versus neutral drinking water on glucose regulation, gut microbiota, or low-grade inflammatory state (89).

The discrepancy between studies (89, 96) may reflect differences in trial duration, water composition, and participant baseline oxidative status. Differences in baseline oxidative status between study populations may have contributed to the variable findings (96). The potential influence of baseline oxidative stress is further supported by the modest improvements observed in participants with metabolic syndrome, whose higher initial oxidative burden may have allowed greater measurable change. Differences in marker sensitivity may also have contributed. For example, 8 hydroxy 2 deoxyguanosine is considered a more sensitive marker of oxidative DNA damage, whereas malondialdehyde and cytokines can be more variable and less stable, which may partly explain the inconsistency in results.

Overall, although the mechanistic hypotheses appear promising, the evidence supporting alkaline water as a therapeutic or clinically useful immunomodulatory intervention remains speculative. No human trial demonstrated a strong immune related clinical endpoint, highlighting the lack of translational support for claims of significant immunological benefit. For the same reasons discussed in the metabolism section, animal model findings cannot be directly extrapolated to humans. Therefore, while alkaline water may show antioxidant activity under certain conditions, claims of immune enhancement remain inconclusive. Further research using standardized protocols and well controlled study designs is needed to better determine its potential therapeutic role. A summary of the immunological and oxidative stress outcomes of alkaline water interventions is shown in Table 7.

**Table 7:** Immunological and oxidative stress outcomes of alkaline water interventions in human and animal studies.

Directionality	Population/model	Design	Intervention	Duration	Outcomes	Key findings	References
+	Adults (MetS)	RCT	High-concentration hydrogen-rich water	24 weeks	lipid profile, glucose markers, inflammatory/redox biomarkers	Improved selected metabolic and inflammatory/redox biomarkers	(88)
0	Healthy young adult men	Randomized cross-over intervention	Alkaline versus neutral drinking water	Two 2-week intervention periods	Gut microbiota, glucose regulation, low-grade inflammatory state	No differential effect compared with neutral water	(89)
+++	Diabetic mouse models	Preclinical animal study	Electrolyzed reduced water	6 weeks in STZ-induced diabetic mice; 29 days in db/db diabetic mice	Blood glucose, insulin, glucose tolerance; proposed ROS-related mechanism	Improved glucose-related outcomes; ROS-scavenging mechanism proposed but not definitive	(92)
+++	Rats (diabetic model)	Animal Experimental Study	Alkaline Reduced Water	~6–8 weeks	TNF-α, IL-6, adiponectin	↓ TNF-α, ↓ IL-6, ↑ adiponectin	(93)
+	Healthy adults	Randomized double-blind placebo-controlled trial	Electrolyzed-reduced water	8 weeks	Oxidative stress biomarkers, biological antioxidant potential, health-related indices	↓ d-ROMs and improved antioxidant potential; surrogate outcomes only	(96)
+	Healthy adults	RCT	Hydrogen-rich water	4 weeks	Oxidative stress biomarkers, inflammatory cytokines	↓ oxidative stress and inflammatory responses compared with placebo	(97)
++	Intestinal epithelial cell/macrophage co-culture model	In vitro experimental study	Alkaline Reduced Water	24 h epithelial oxidative stress exposure + 24 h macrophage exposure	IL-6, IL-10, MCP, TNF-α, RANTES, mitochondrial dysfunction	Reversal/attenuation of oxidative stress-related immunokine changes and mitochondrial dysfunction	(98)

Description: Directionality of outcomes is represented as follows: +++ indicates strong positive effects, ++ moderate positive effects, + weak or modest effects, and 0 no statistically significant effect. d-ROMs, derivatives of reactive oxygen metabolites; db/db, genetically diabetic db/db mouse model; IL-6, interleukin-6; IL-10, interleukin-10; MCP, monocyte chemoattractant protein; MetS, metabolic syndrome; RANTES, regulated upon activation, normal T cell expressed and secreted; RCT, randomized controlled trial; ROS, reactive oxygen species; STZ, streptozotocin; TNF-α, tumor necrosis factor-alpha.

## 7 Future research directions

The current landscape of alkaline water research highlights a critical need for high-quality, translational studies to move beyond anecdotal evidence and speculative biochemistry. Future investigations should prioritize the following areas:

- Standardized Clinical Trials: There is a pressing need for multi-center, double-blind, randomized controlled trials (RCTs) with large, diverse human cohorts. Future studies must define standardized protocols for water alkalinity (pH levels), mineral composition, and administration frequency to ensure reproducibility across different research settings.
- Long-term Longitudinal Studies: Most existing human data are derived from short-term interventions. Long-term longitudinal studies are necessary to evaluate the physiological and clinical risks associated with the prolonged consumption of high-pH water, particularly regarding its impact on gastric acidity, nutrient absorption (e.g., Vitamin B12 and iron), and renal function.
- Mechanistic Multi-Omics Analysis: To move beyond speculative “immunomodulatory” claims, future research should employ multi-omics approaches, including metagenomics and metabolomics; to examine the influence of alkaline water on the human gut microbiota and metabolic signaling pathways (e.g., the Nrf2 and NF-κB pathways).
- Isolation of Active Agents: Recent literature suggests that the therapeutic potential of electrolyzed-reduced water (ERW) may be attributed to dissolved molecular hydrogen (H_2_) rather than alkalinity itself. Future research should include comparative arms; alkaline water vs. neutral hydrogen-rich water, to isolate the specific chemical drivers of observed antioxidant effects.
- Targeted Clinical Populations: Rather than broad “wellness” applications, research should focus on specific clinical conditions where alkalinity or hydrogen content may offer adjunctive benefits, such as hyperuricemia (uric acid regulation), acid reflux (pepsin inactivation), or exercise-induced metabolic acidosis.
- Safety and Adverse Effects: Rigorous safety profiles must be established, specifically for potential “alkaline-alkali syndrome” or disturbances in the body’s natural buffering systems in vulnerable populations, such as the elderly or those with (CKD).

## 8 Overall conclusion

In light of the considerations presented in this review, the scientific validity of alkaline water as a therapeutic intervention remains largely unsubstantiated. While the mechanistic hypotheses regarding its antioxidant and buffering capacities appear promising in *in vitro* and preliminary animal models, the current body of peer-reviewed literature fails to provide robust evidence for clinical efficacy in humans. Specifically, the lack of human trials demonstrating strong immune-related clinical endpoints highlights a significant disconnect between laboratory theory and translational medicine. As discussed, the physiological complexity of human pH homeostasis suggests that findings from simplified animal models cannot be directly extrapolated to human clinical outcomes. Consequently, while alkaline water may exhibit localized antioxidant activity under specific conditions, its purported role as a significant immunomodulatory or health-enhancing agent remains speculative. The current popularity of alkaline water likely reflects the intersection of persuasive marketing strategies and the perennial appeal of simple solutions to complex biological processes, rather than a foundation of rigorous clinical data. Therefore, the health benefits of alkaline water are currently categorized more as a modern wellness trend than a proven medical intervention. Future research utilizing standardized protocols, longitudinal designs, and well-controlled human cohorts is essential to definitively determine whether alkaline water possesses a genuine therapeutic role or if its benefits are primarily anecdotal.
